# 2427: Metabo-therapy for RARRES1-depleted epithelial cells using repurposed mitochondrial metabolism inhibitor, metformin

**DOI:** 10.1017/cts.2017.49

**Published:** 2018-05-10

**Authors:** Sara Maimouni, Mi-Hye Lee, Stephen Byers

**Affiliations:** Georgetown - Howard Universities, Washington, DC, USA

## Abstract

OBJECTIVES/SPECIFIC AIMS: The goal of this study is to examine bioenergetic phenotype of retinoic acid receptor responder 1 (RARRES1)-depleted epithelial cells and to facilitate the discovery of personalized metabo-therapeutics in the context of cancers characterized with loss of or low expression of RARRES1. METHODS/STUDY POPULATION: Anoikis assay and annexinV labeling were used to assess drug resistance and apoptotic phenotype in RARRES1-depleted epithelial cells. Metabolomics, AMP kinase activity, mito-tracker, and extracellular flux assays were used to examine the bioenergetic profile of RARRES1-depleted epithelial cells. Extracellular flux assays were used to assess the phenotype of RARRES1-depleted epithelial cells treated with or without metformin. RESULTS/ANTICIPATED RESULTS: RARRES1 is a major regulator of mitochondrial function. Its depletion in tumors induces an oxidative phosphorylation dependent phenotype and subsequently increases ATP abundance in the cell, enhances anabolic pathways and increases survival. Treatment with FDA approved mitochondrial respiration inhibitor, metformin, reversed the metabolic phenotype of RARRES1 depleted-epithelial cells. Metformin could be the ideal therapeutics to reduce tumor burden in cancers with loss of or low expression of RARRES1. DISCUSSION/SIGNIFICANCE OF IMPACT: Bioenergetic dynamics are emerging as a basis for understanding the pathology of cancer. The malignancy progresses as its metabolic pattern and mitochondrial respiration become more dysfunctional. The regulatory pathways of bioenergetic dynamics are currently poorly understood, and the characterization of proteins implicated in those processes must be assessed. One understudied protein and tumor suppressor is RARRES1. RARRES1 is induced by retinoic acid (a major metabolic regulator) and functions as a putative carboxypeptidase inhibitor. Understanding the connection between this carboxypeptidase inhibitor and intermediary metabolism will enlighten our understanding of the bioenergetic profile of cells and facilitate the discovery of personalized metabo-therapeutics in the context of cancer.

